# The Effect of Intraoperative Fluid Management According to Stroke Volume Variation on Postoperative Bowel Function Recovery in Colorectal Cancer Surgery

**DOI:** 10.3390/jcm10091857

**Published:** 2021-04-25

**Authors:** Ki-Young Lee, Young-Chul Yoo, Jin-Sun Cho, Wootaek Lee, Ji-Young Kim, Myoung-Hwa Kim

**Affiliations:** 1 Department of Anesthesiology and Pain Medicine, Anesthesia and Pain Research Institute, Yonsei University College of Medicine, 50-1 Yonsei-ro, Seodaemun-gu, Seoul 03722, Korea; KYLEE504@yuhs.ac (K.-Y.L.); seaoyster@yuhs.ac (Y.-C.Y.); CHJS0214@yuhs.ac (J.-S.C.); WOOTAEK0513@yuhs.ac (W.L.); AMYJYKIM@yuhs.ac (J.-Y.K.); 2 Department of Anesthesiology and Pain Medicine, Gangnam Severance Hospital, 211 Eonju-ro, Gangnam-gu, Seoul 06273, Korea

**Keywords:** colon cancer surgery, goal-directed fluid therapy, recovery, bowel movement, stroke volume variation

## Abstract

Stroke volume variation (SVV) has been used to predict fluid responsiveness; however, it remains unclear whether goal-directed fluid therapy using SVV contributes to bowel function recovery in abdominal surgery. This prospective randomized controlled trial aimed to compare bowel movement recovery in patients undergoing colon resection surgery between groups using traditional or SVV-based methods for intravenous fluid management. We collected data between March 2015 and July 2017. Bowel function recovery was analyzed based on the gas-passing time, sips of water time, and soft diet (SD) time. Finally, we analyzed data from 60 patients. There was no significant between-group difference in the patients’ characteristics. Compared with the control group (*n* = 30), the SVV group (*n* = 30) had a significantly higher colloid volume and lower crystalloid volume. Moreover, the gas-passing time (77.8 vs. 85.3 h, *p* = 0.034) and SD time (67.6 vs. 85.1 h, *p* < 0.001) were significantly faster in the SVV group than in the control group. Compared with the control group, the SVV group showed significantly lower scores of pain on a numeric rating scale and morphine equivalent doses during post-anesthetic care, at 24 postoperative hours, and at 48 postoperative hours. Our findings suggested that, compared with the control group, the SVV group showed a faster postoperative SD time, reduced acute postoperative pain intensity, and lower rescue analgesics. Therefore, SVV-based optimal fluid management is expected to potentially contribute to postoperative bowel function recovery in patients undergoing colon resection surgery.

## 1. Introduction

Traditionally, fluids are administered during intestinal resection based on several factors, including preoperative fasting time, intraoperative bleeding, blood pressure maintenance, and loss of one-third volume of water. In cases with a wide resection range or a long surgical duration, hypovolemia may reduce tissue perfusion and causes detrimental organ dysfunctions. However, excessive intraoperative fluid administration also leads to poor outcomes, including increased postoperative bowel edema, respiratory complications, and longer durations of hospital stay. Therefore, goal-directed fluid supply is warranted since optimal intraoperative supply of fluids improves postoperative recovery and prognosis [1,2,3]. Unfortunately, optimal fluid replacement therapy during major abdominal surgery remains unclear.

During the perioperative period, there are possible negative or positive interactions between fluid therapy and gastrointestinal (GI) function. Inadequate fluid management may cause delayed GI function recovery and may thus prevent early oral intake. Moreover, postoperative GI dysfunction may cause electrolyte loss and metabolic problems [3]. For precise measurement of adequate intravascular volume, central venous pressure, pulmonary artery occlusion pressure, the intrathoracic blood volume index, and the left ventricular end-diastolic volume index were introduced; however, these often fail to provide reliable information or to predict fluid responsiveness and often provide conflicting reports [4,5,6]. Consequently, viewing the fluid response through SVV after catheter insertion through the radial artery is a more widely used method [7,8]. Stroke volume variation (SSV) refers to differences in stroke volume during the respiratory cycle. It can be indirectly measured through arterial pressure waveform analysis on an EV1000™ monitor using the Flo Trac Vigilo^TM^. SVV has been performed to predict the degree of fluid responsiveness in abdominal surgery [9,10]. In addition, Asklid D et al. reported that goal-directed fluid management improves long-term survival after colorectal cancer surgery [11]. On the other hand, a separate meta-analysis concluded that goal-directed fluid management does not improve postoperative GI function recovery [12]. Therefore, the utility of SVV in improving postoperative bowel movement recovery remains unclear.

We hypothesized that intraoperative goal-directed fluid management could maintain the optimal intravascular volume status in patients with abdominal surgery and could subsequently help recover postoperative bowel function. Accordingly, we aimed to compare SVV-based and conventional fluid management in terms of bowel movement recovery after colorectal cancer surgery.

## 2. Materials and Methods

### 2.1. Study Population

This was a single-center, prospective, randomized controlled clinical trial. The protocol was approved by the ethical standards of the Severance Hospital Research Ethics Committee and Institutional Review Board (IRB number: 4-2014-0730); moreover, it was registered at clinicalTrials.gov (NCT02288767). This study was conducted according to the World Medical Association Declaration of Helsinki. All patients provided written informed consent before study enrolment. Using the American Society of Anesthesiologists’ physical status Ⅰ–Ⅲ, we assessed adult patients who underwent elective colorectal cancer surgery at our tertiary hospital between March 2015 and August 2017 for eligibility. The exclusion criteria were an unexpected surgical plan change, consent retraction, a preoperative left ventricular ejection fraction <40%, significant cardiac arrhythmias or valve regurgitation, coagulation disorders (activated partial thromboplastin time >1.5 times normal value), preoperative renal insufficiency (serum creatinine >2 mg/dL, oliguria, anuria, or hemodialysis), impaired hepatic function (phosphatase alkaline, aspartate aminotransferase, and alanine aminotransferase >2 times normal values), preoperative infection, current pregnancy, and participation in another trial.

A computer-generated randomization table was used to randomly allocate patients to either the SVV group (patients undergoing intraoperative fluid management with SVV guidance) or the control group (patients undergoing conventional intraoperative fluid management) in a 1:1 ratio, and copies of the random sequence were kept in sealed, opaque envelopes. Randomization was not blocked or stratified. Only the investigator who performed the intervention in the operating room was aware of the allocation group, with the patients and other outcome researchers being blinded to the group allocation.

Upon the patient’s arrival to the operating room, an electrocardiogram and pulse oximeter were attached, followed by noninvasive blood pressure measurement. For anesthesia induction, 1.5–2.0 mg/kg of propofol was injected with continuous remifentanil infusion at 0.2 mcg/kg/min. After the administration of 0.8 mg/kg of rocuronium for muscle relaxation, endotracheal intubation was performed. Depending on the surgery type and scope, additional venous catheterization was established; moreover, monitoring of blood pressure and laboratory tests were performed with arterial catheterization. Based on the assigned group, the patients underwent or did not undergo SVV monitoring using the FloTrac/EV1000™ monitor. Anesthesia was maintained using 50% oxygen, desflurane, remifentanil, and rocuronium. To determine the appropriate anesthesia depth, the bispectral index (BIS) was measured and maintained at a level of 40–60. Additionally, the remifentanil infusion rate was adjusted within 0.05 to 0.5 mcg/kg/min to maintain the intraoperative blood pressure and pulse rate within 20% of their basal values.

Intraoperative fluid management was performed based on the assigned group. Depending on the patient, the fluids were administered based on the preoperative fasting time, intraoperative fluid loss (evaporation, shedding, urination, surgery site, etc.), and expected blood loss. The intraoperatively maintained fluid was crystalloid in both groups at about 4 mL/kg/h. In the SVV group, bolus colloid (single bolus: 100–200 mL, maximal 30 mL/kg) was administered when SVV >12%, but crystalloid or colloid was empirically administered in the control group. The main crystalloid was lactate linger solution, and the colloid was hydroxyethyl starch (HES 130/0.4). In case hypotension (MBP falling to less than 65 mmHg within 5 min) occurred for SVV <12%, a vasoconstrictor was intermittently injected or continuously infused. In the control group, the crystalloid and colloid (max: 30 mL/kg) were administered based on conventional parameters: blood pressure, heart rate, output volume, and the anesthesiologist’s preference. Since mechanical respiration is a confounding factor of SVV, a tidal volume of 8 mL/kg and the positive end-expiratory pressure (PEEP) were unified as ‘0′. Thirty minutes before the expected end of the surgery, 1.0 g of propacetamol and 0.075 mg of palonosetron were intravenously infused for postoperative pain control and nausea prevention, respectively. At operation end, desflurane and remifentanil administration were terminated; furthermore, muscle relaxation was evaluated through a train-of-four test using a nerve stimulator and reversed using 0.2 mg of glycopyrrolate and 1.0 mg of neostigmine. After extubation, the patients were transferred to the post-anesthetic care room (PACU).

During PACU stay, pain was evaluated using a 10-point numeric rating scale; moreover, the rescue analgesic consumption was recorded as the morphine equivalent dose. Furthermore, we assessed the patient’s nausea incidence and consumed antiemetics. In addition, follow-up measurements of the pain intensity score as well the consumption of rescue analgesics and antiemetics were obtained at 6, 24, and 48 postoperative hours. The sips of water (S.O.W), gas-passing, and soft diet times were used to analyze bowel function recovery. Moreover, we evaluated the occurrence and types of postoperative complications, whether the patient was transferred to the intensive care unit (ICU), the number of days of postoperative hospital stay, and whether the patient died within the study period (from March 2015 and August 2017).

### 2.2. Statistical Analyses

A previous study [13] suggested that the gas-passing time of patients undergoing colorectal resection surgery was approximately 64 h. Based on the alpha, power, and drop-out rates of 0.05, 90%, and 15%, respectively, the number of participants per group was 30; consequently, the total sample size was 60 participants. Our primary outcome was to investigate the postoperative GI function recovery using the gas-passing time, and the SD and S.O.W start times. The secondary outcomes were other postoperative recovery profiles such as postoperative pain and nausea, surgical complications, and length of stay. At a significance level of 5%, all hypotheses underwent two-tailed testing. All continuous and nominal variables were expressed as mean ± standard deviation (or median [IQR]) and *n* (%), respectively. After the Kolmogorov–Smirnov and Shapiro–Wilk tests for normality assumption, continuous and categorical variables were analyzed using the Student *t*-test (or Mann–Whitney U nonparametric test) and Fisher’s exact test, respectively. Statistical significance was set at *p* < 0.05. All statistical analyses were performed using SPSS (SPSS version 23.0; IBM Corp., Armonk, NY, USA).

## 3. Results

**Figure 1:**
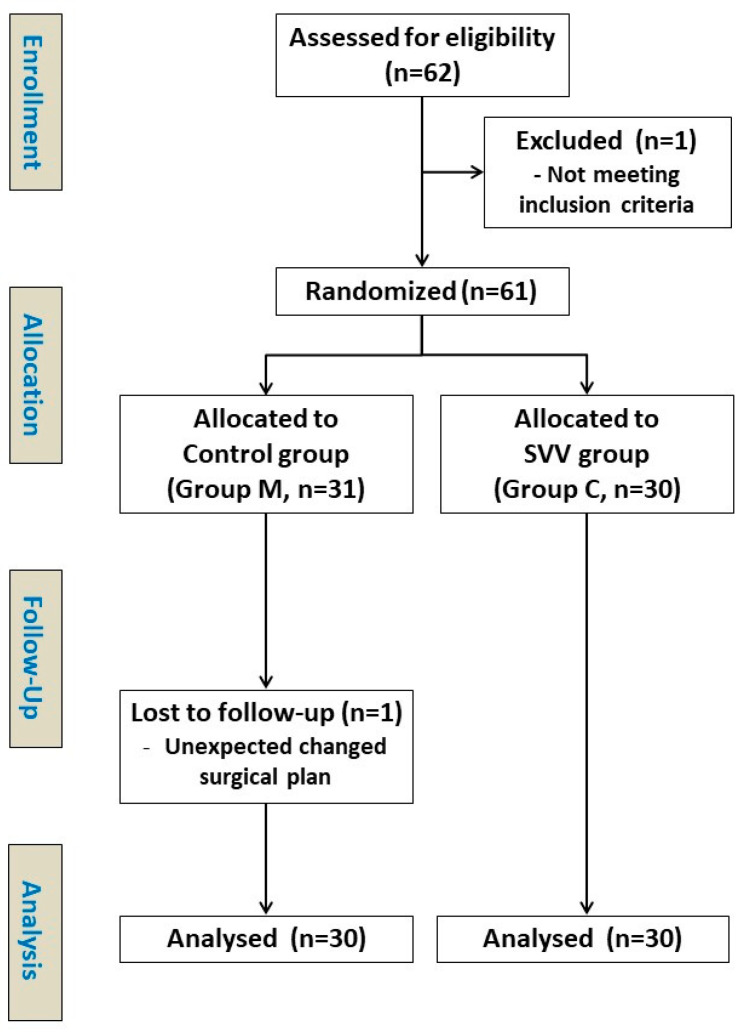
CONSORT diagram.

We enrolled 62 patients who underwent colorectal cancer surgery between March 2015 and August 2017. One patient did not meet the inclusion criteria, while another patient in the control group dropped out after randomization due to an unexpected surgical plan change; finally, 60 patients were analyzed (Figure 1).

Table 1 shows the patient demographic data. There were no significant between-group differences in age, gender, physical status, comorbidities, surgical procedures, surgical duration, and patent-controlled analgesia type.

Table 2 lists the intraoperative parameters, including fluid management. There were no significant between-group differences in the blood test results, including vital signs, of in the number of patients who were intraoperatively administered with vasoconstrictors, in the amount of bleeding, and in urine output. However, there were significant between-group differences in the amounts of colloid (851.2 ± 461.8 mL vs. 616.7 ± 313.0 mL, *p* < 0.001) and crystalloid (806.0 ± 898.6 mL vs. 1615.8 ± 814.8 mL, *p* = 0.033) administered.

Table 3 presents the postoperative pain intensity and consumption of rescue analgesics, including antiemetics. In the post-anesthesia recovery room, the SVV group showed significantly lower pain intensity scores than the control group (3.3 ± 1.1 vs. 4.3 ± 1.2, *p* = 0.001). Additionally, the pain intensity scores at 6, 24, and 48 postoperative hours were significantly lower in the SVV group (3.3, 3.8, and 4.1) than in the control group (4.3, 4.5, and 5.1). Furthermore, the SVV group showed lower rescue analgesic consumption up to 24 h than the control group (*p* = 0.025). There was no significant between-group difference in the number of patients receiving antiemetics.

Table 4 demonstrates the results of our primary endpoint. Compared with the control group, the SVV group showed a significantly faster soft diet time (67.6 ± 17.1 h vs. 85.1 ± 16.8 h, *p* = 0.034) and gas-passing time (77.8 ± 36.6 h vs. 85.3 ± 18.5 h, *p* < 0.001). The most common postoperative complication was fever, which was controlled using antipyretics and ice bag application. Intra-abdominal infections in two patients were treated using antibiotic management; moreover, pneumonia and asthma-like respiratory complications in two patients subsided after supplying nasal O_2_ and using a nebulizer. Regarding cardiovascular problems, one patient had suspected ischemic heart disease based on an electrocardiogram; however, it was checked at a post-discharge follow-up and did not present any events. Regarding wound problems, one patient showed seroma formation, which was controlled through wound dressing and antibiotic management. One patient in each group presented postoperative ileus, with that in the SVV group being surgically resolved. One patient with suspected anastomosis site leakage and another with dyspepsia spontaneously recovered and were discharged from the hospital. There were four patients in the control group with postoperative acute kidney injury based on the “RIFLE (Risk, Injury, Failure, Loss, and End stage) criteria, which spontaneously recovered through hydration. There was no between-group difference in the postoperative hospital stay duration (average eight days); moreover, three patients in the control group were transferred to the ICU while one patient died in each group within the study period. Overall, there were no significant between-group differences in the incidence of postoperative complications.

## 4. Discussion

This study evaluated the utility of SVV for intraoperative fluid management in patients undergoing colorectal cancer surgery by comparing prognosis, including intestinal motility recovery and surgical complications. Compared with the control group, the SVV group showed significantly faster gas-passing and soft diet start times. In contrast to previous studies assessing abdominal surgery, we only targeted patients with colorectal cancer surgery and minimized the specific surgery effects; moreover, we focused on postoperative clinical recovery, with bowel movement recovery as our primary endpoint. Since colorectal surgery patients may present acute alterations in volume status, precise fluid and electrolyte administration is critical for their overall perioperative management, which critically affects postoperative morbidity and mortality. Perioperative fluid management seeks to achieve a balance between avoiding hypotension, impaired tissue oxygenation, and inadequate organ profusion, which may be associated with fluid depletion, and avoiding interstitial edema and cardiopulmonary complications related to fluid overload. Although the basic goals of perioperative fluid management have been established, how to attain these goals remains unclear.

As an alternative to static variables, SVV has been used as a hemodynamic indicator for predicting fluid responsiveness in patients undergoing mechanical ventilation [14,15]. Accordingly, arterial pulse waveform analysis has been proposed to monitor both cardiac output (CO) and SVV [16,17]. Regarding echocardiography, it is difficult to manipulate the probe and to read the screen, which leads to professional human effort being required, which is a disadvantage. Therefore, a more common approach is fluid response monitoring through SVV after catheter insertion into the radial artery [7,8]. Generally, SVV is used to predict fluid responsiveness in patients who are mechanically ventilated. Mechanical ventilation induces cyclic variations in cardiac preload, which are reflected by cyclic changes in systolic arterial pressure, arterial pulse pressure, and left ventricular stroke volume [18]. Although ventilation-related cyclical changes do not induce CO changes in inadequately filled patients, they can induce significant changes with hypovolemia. Patients with high SVV values are essentially on the steep portion of Frank Starling’s curve, which plots the preload effects on stroke volume. SVV is associated with ventilation, with larger fluctuations being observed in hypovolemia. To minimize these respiratory effects on SVV, we did not permit spontaneous respiration using muscle relaxant administration and using a ventilator mode of volume control with PEEP set to 0.

Maintaining the optimal volume state is among the important factors for maintaining favorable organ perfusion and optimal oxygen supply, which requires accurate volume state evaluation and prediction. Intraoperative measurements of the effective circulating blood volume can be obtained using parameters such as blood loss, urine output, third space, and insensible losses; however, they only serve as a rough estimate [1]. Patients undergoing major abdominal surgery are recommended to undergo preoperative crystalloid loading at 2 mL/kg/h of fasting, with subsequent crystalloid infusion at three to four times the actual blood loss. Furthermore, the patients frequently received 4 to 8 mL/kg/h of the crystalloid based on suspected insensible losses, including third spacing and evaporation. This could lead to basal crystalloid infusion rates of up to 20 mL/kg/h, which are frequently titrated to yield urine outputs of 0.5 to 1 mL/kg/h [19]. However, this is limited since it is insensitive to adequate fluid supply based on the blood vessel state. To optimize the patient’s blood volume, the effective circulating blood volume should be properly intraoperatively supplied to balance the blood volume, urine volume, third space, and insensible loss. Regarding central venous or pulmonary arterial pressure measurement through the central venous catheter, fluids can be supplied while monitoring cardiovascular changes and the blood volume status. However, it cannot be applied to all patients given its invasiveness; moreover, numerous reports have indicated that venous pressure does not sufficiently reflect blood status [1,20].

There is accumulating evidence showing that intraoperative fluid administration may affect patient outcomes after major surgery [21]. Specifically, the fluid quantity administered significantly influences the incidence of postoperative complications [22,23]. A restrictive fluid strategy contributes to better major-surgery outcomes; furthermore, goal-directed therapy fluid supply has been shown to improve the outcomes. However, excessive fluid restriction involving hypovolemia may cause organ dysfunction, increased postoperative morbidity, and death [24]. This is difficult to determine given the large inter-practitioner variations in fluid management approaches, which has led to a wide variation in patient care [25,26]. To mitigate this variation, goal-directed fluid therapy based on optimizing flow-related variables has been demonstrated as the best approach for fluid administration in high-risk surgical patients [27,28]. Unfortunately, there has been low and slow adoption of these strategies by providers and institutions. Among the challenges impeding implementation is the need for substantial training and vigilance in applying goal-directed fluid therapy strategies. Even in the study conditions, there is suboptimal compliance with treatment protocols [29,30]. Several recent studies have confirmed the positive effects of using a goal-directed fluid protocol for guiding fluid administration [31,32]. Although this strategy has been recommended by professional societies in European countries [33], it is not commonly implemented in clinical practice [34].

This study has several limitations. First, this was a single-center small-scale study; therefore, there is a need for studies with larger sample sizes to confirm our findings. Additionally, in laparoscopic surgery, changes in cardiovascular status may affect the SSV values; therefore, SSV being only applied for open surgeries is a limitation. However, recently, laparoscopic surgery has become relatively accurate; moreover, the current trend in surgical procedures is leaning towards laparoscopy or robot-assisted surgery rather than open abdominal surgery. Therefore, this could be also considered a strength of our study. Third, the pain intensity and the amount of rescue analgesics after surgery, which are important factors influencing bowel movement, were higher in the control group compare with the SVV group and can be regarded as a bias that may affect the delay in GI function in addition to intraoperative fluid therapy.

## 5. Conclusions

In conclusion, compared with the control group, the SVV group presented faster postoperative soft diet times, which were among the primary objectives regarding the recovery of bowel function after bowel resection surgery. Furthermore, the SVV group showed reduced postoperative pain intensity and consumption of rescue analgesics than the control group.

## Figures and Tables

**Table 1:** Demographic data.

	Control Group (N = 30)	SVV Group (N = 30)	*p*-Value
Age (year)	57.9 (6.1)	60.3 (8.9)	0.077
Gender (M/F)	17 (13)	24 (6)	0.095
Height (cm)	163.0 (9.3)	164.6 (6.7)	0.722
Weight (kg)	63.6 (15.5)	65.5 (12.8)	0.829
ASA physical status			0.713
1	12	9	
2	12	15	
3	6	6	
Comorbidity			
Hypertension	12	14	0.795
Diabetes Mellitus	3	4	>0.999
Cardiovascular	4	1	0.353
Respiratory	5	5	>0.999
Neurologic	1	0	>0.999
Liver	0	1	>0.999
Kidney	1	4	0.353
Anesthetic duration (min)	285 (66.5)	261.2 (88.9)	0.074
Surgical duration (min)	239.3 (59.1)	218.2 (83.3)	0.056
Surgical types			0.360
Laparoscopic	21	25	
Open	9	5	
Surgical procedures			0.691
Right hemicolectomy	7	8	
Anterior resection	9	12	
Low anterior resection	13	8	
Left hemicolectomy	0	1	
Total colectomy	1	1	
Patient-controlled analgesia (via)			>0.999
Intravenous	27	27	
Epidural	3	3	

**Table 2:** Intraoperative parameters.

	Control Group (N = 30)	SVV Group (N = 30)	*p*-Value
Initial vital signs			
HR (BPM)	71.6 (11.8)	70.3 (11.3)	0.834
MBP (mmHg)	82.4 (13.7)	86.5 (12.8)	0.181
SpO_2_ (%)	100 (0)	100 (0)	>0.999
Vasopressor administration	15	15	>0.999
Ephedrine	7	8	
Phenylephrine	7	7	
Norepinephrine	1	0	
Bradycardia event	1	0	>0.999
Laboratory values at baseline			
PH	7.443 (0.03)	7.435 (0.04)	0.315
PO_2_	194.0 (43.4)	194.1 (54.3)	0.806
PCO_2_	33.3 (4.8)	34.6 (4.0)	0.126
Hematocrit	11.3 (1.6)	11.6 (1.5)	0.246
Lactate	0.83 (0.2)	0.80 (0.2)	0.414
Laboratory values at surgery end			
PH	7.428 (0.10)	7.401 (0.04)	0.305
PO_2_	175 (31.5)	184.1 (31.1)	0.265
PCO_2_	34.9 (3.8)	37.1 (4.4)	0.054
Hematocrit	11.3 (1.4)	11.4 (1.4)	0.777
Lactate	0.90 (0.3)	0.85 (0.2)	0.419
Input			
Crystalloid (mL)	1615.8 (814.8)	806.0 (898.6)	0.033
Colloid (mL)	616.7 (313.0)	851.2 (461.8)	<0.001
RBC (mL)	41.7 (160.9)	16.7 (57.7)	0.901
Output			
Urine (mL)	397.0 (311.1)	459.8 (403.7)	0.545
Bleeding (mL)	159.0 (278.1)	93.3 (182.7)	0.078

**Table 3:** Postoperative pain and emesis.

	Control Group (N = 30)	SVV Group (N = 30)	*p*-Value
Post-Anesthetic Care Unit			
Numeric Rating Scale (Pain)	4.3 (1.2)	3.3 (1.1)	0.001
Morphine equivalent dose (mg)	32.2 (38.8)	16.7 (27.3)	0.108
Antiemetic requirement	1	0	0.483
Stay duration (min)	45.1 (11.7)	40.0 (10.5)	0.077
Postoperative 1–6 h			
Numeric Rating Scale (Pain)	4.5 (1.1)	3.8 (1.0)	0.033
Morphine equivalent dose (mg)	60.0 (46.8)	46.7 (41.4)	0.285
Antiemetic requirement	14	20	0.192
Postoperative 6–24 h			
Numeric Rating Scale (Pain)	5.1 (1.7)	4.1 (1.5)	0.018
Morphine equivalent dose (mg)	101.5 (86.6)	56.7 (58.3)	0.025
Antiemetic requirement	15	19	0.435
Postoperative 24–48 h			
Numeric Rating Scale (Pain)	4.2 (1.4)	3.0 (1.1)	0.002
Morphine equivalent dose (mg)	61.7 (71.5)	28.3 (40.9)	0.061
Antiemetic requirement	2	1	>0.999

**Table 4:** Postoperative recovery profile.

	Control Group (N = 30)	SVV Group (N = 30)	*p*-Value
Bowel movement recovery			
Sips of water time (h)	42.4 (11.6)	37.3 (11.7)	0.051
Soft diet time (h)	85.1 (16.8)	67.6 (17.1)	<0.001
Gas passing time (h)	85.3 (18.5)	77.8 (36.6)	0.034
Postoperative Complications			
Total cases	15	22	0.110
Fever (>38 °C)	12	20	0.069
Intra-abdominal infection	1	1	>0.999
Respiratory	0	2	>0.999
Cardiovascular	0	1	>0.999
Ileus	1	1	>0.999
Wound problem	1	0	>0.999
Anastomosis leakage	0	1	>0.999
Dyspepsia	0	1	>0.999
Kidney injury	4	0	0.112
Reoperation	0	1	>0.999
Postoperative length of stay in hospital	8.1 (5.7)	7.9 (3.8)	0.925
Postoperative intensive care unit transfer	3	0	0.237
Death	1	1	>0.999

## Data Availability

The data presented in this study are available on request from the corresponding author. The data are not publicly available due to privacy.

## References

[1] Bamboat Z.M., Bordeianou L. (2009). Perioperative fluid management. Clin. Colon Rectal Surg..

[2] Bleier J.I., Aarons C.B. (2013). Perioperative fluid restriction. Clin. Colon Rectal. Surg..

[3] Patel S., Lutz J.M., Panchagnula U., Bansal S. (2012). Anesthesia and perioperative management of colorectal surgical patients—Specific issues (part 2). J. Anaesthesiol. Clin. Pharmacol..

[4] Feissel M., Mangin I., Ruyer O., Faller J.-P., Michard F., Teboul J.-L. (2001). Respiratory changes in aortic blood velocity as an indicator of fluid responsiveness in ventilated patients with septic shock. Chest.

[5] Bendjelid K., Romand J.-A. (2003). Fluid responsiveness in mechanically ventilated patients: A review of indices used in intensive care. Intensive Care Med..

[6] Michard F., Teboul J.L. (2002). Predicting fluid responsiveness in icu patients: A critical analysis of the evidence. Chest.

[7] Lahner D., Kabon B., Marschalek C., Chiari A., Pestel G., Kaider A., Fleischmann E., Hetz H. (2009). Evaluation of stroke volume variation obtained by arterial pulse contour analysis to predict fluid responsiveness intraoperatively. Br. J. Anaesthesiol..

[8] Derichard A., Robin E., Tavernier B., Costecalde M., Fleyfel M., Onimus J., Lebuffe G., Chambon J.-P., Vallet B. (2009). Automated pulse pressure and stroke volume variations from radial artery: Evaluation during major abdominal surgery. Br. J. Anaesthesiol..

[9] Zhang Z., Lu B., Sheng X., Jin N. (2011). Accuracy of stroke volume variation in predicting fluid responsiveness: A systematic review and meta-analysis. J. Anesth..

[10] Li C., Lin F.-Q., Fu S.-K., Chen G.-Q., Yang X.-H., Zhu C.-Y., Zhang L.-J., Li Q. (2013). Stroke volume variation for prediction of fluid responsiveness in patients undergoing gastrointestinal surgery. Int. J. Med. Sci..

[11] Asklid D., Segelman J., Gedda C., Hjern F., Pekkari K., Gustafsson U. (2017). The impact of perioperative fluid therapy on short-term outcomes and 5-year survival among patients undergoing colorectal cancer surgery—A prospective cohort study within an ERAS protocol. Eur. J. Surg. Oncol. (EJSO).

[12] Xu C., Peng J., Liu S., Huang Y., Guo X., Xiao H., Qi D. (2018). Goal-directed fluid therapy versus conventional fluid therapy in colorectal surgery: A meta analysis of randomized controlled trials. Int. J. Surg..

[13] Kim M.H., Lee K.Y., Lee K.Y., Min B.S., Yoo Y.C. (2016). Maintaining optimal surgical conditions with low insufflation pressures is possible with deep neuromuscular blockade during laparoscopic colorectal surgery: A prospective, randomized, double-blind, parallel-group clinical trial. Medicine (Baltimore).

[14] Rex S., Brose S., Metzelder S., Hüneke R., Schälte G., Autschbach R., Rossaint R., Buhre W. (2004). Prediction of fluid responsiveness in patients during cardiac surgery. Br. J. Anaesthesiol..

[15] Marx G., Cope T., McCrossan L., Swaraj S., Cowan C., Mostafa S.M., Wenstone R., Leuwer M. (2004). Assessing fluid responsiveness by stroke volume variation in mechanically ventilated patients with severe sepsis. Eur. J. Anaesthesiol..

[16] Opdam H.I., Wan L., Bellomo R. (2007). A pilot assessment of the flotrac cardiac output monitoring system. Intensive Care Med..

[17] Penttilä J., Snapir A., Kentala E., Koskenvuo J., Posti J., Scheinin M., Scheinin H., Kuusela T. (2006). Estimation of cardiac output in a pharmacological trial using a simple method based on arterial blood pressure signal waveform: A comparison with pulmonary thermodilution and echocardiographic methods. Eur. J. Clin. Pharmacol..

[18] Michard F. (2005). Changes in arterial pressure during mechanical ventilation. Anesthesiology.

[19] Campbell I.T., Baxter J.N., Tweedie I.E., Taylor G.T., Keens S.J. (1990). Iv fluids during surgery. Br. J. Anaesthesiol..

[20] Joosten A., Delaporte A., Ickx B., Touihri K., Stany I., Barvais L., Van Obbergh L., Loi P., Rinehart J., Cannesson M. (2018). Crystalloid versus colloid for intraoperative goal-directed fluid therapy using a closed-loop system: A randomized, double-blinded, controlled trial in major abdominal surgery. Anesthesiology.

[21] Myburgh J.A. (2014). Fluid resuscitation in acute medicine: What is the current situation?. J. Intern. Med..

[22] Brandstrup B., Tønnesen H., Beier-Holgersen R., Hjortsø E., Ørding H., Lindorff-Larsen K., Rasmussen M.S., Lanng C., Wallin L., Iversen L.H. (2003). Effects of intravenous fluid restriction on postoperative complications: Comparison of two perioperative fluid regimens: A randomized assessor-blinded multicenter trial. Ann. Surg..

[23] Nisanevich V., Felsenstein I., Almogy G., Weissman C., Einav S., Matot I. (2005). Effect of intraoperative fluid management on outcome after intraabdominal surgery. Anesthesiology.

[24] Kumar L., Rajan S., Baalachandran R. (2016). Outcomes associated with stroke volume variation versus central venous pressure guided fluid replacements during major abdominal surgery. J. Anaesthesiol. Clin. Pharmacol..

[25] Lilot M., Ehrenfeld J.M., Lee C., Harrington B., Cannesson M., Rinehart J. (2015). Variability in practice and factors predictive of total crystalloid administration during abdominal surgery: Retrospective two-centre analysis †. Br. J. Anaesthesiol..

[26] Thacker J.K., Mountford W.K., Ernst F.R., Krukas M.R., Mythen M.M. (2016). Perioperative fluid utilization variability and association with outcomes: Considerations for enhanced recovery efforts in sample us surgical populations. Ann. Surg..

[27] Hamilton M.A., Cecconi M., Rhodes A. (2011). A systematic review and meta-analysis on the use of preemptive hemodynamic intervention to improve postoperative outcomes in moderate and high-risk surgical patients. Anesth. Analg..

[28] Pearse R.M., Harrison D.A., MacDonald N., Gillies M.A., Blunt M., Ackland G., Grocott M.P., Ahern A., Griggs K., Scott R. (2014). Effect of a perioperative, cardiac output-guided hemodynamic therapy algorithm on outcomes following major gastrointestinal surgery: A randomized clinical trial and systematic review. JAMA.

[29] Miller T.E., Roche A.M., Gan T.J. (2011). Poor adoption of hemodynamic optimization during major surgery: Are we practicing substandard care?. Anesth. Analg..

[30] Joosten A., Alexander B., Delaporte A., Lilot M., Rinehart J., Cannesson M. (2015). Perioperative goal directed therapy using automated closed-loop fluid management: The future?. Anestezjol. Intensive Ter..

[31] Goepfert M.S., Richter H.P., Eulenburg C.Z., Gruetzmacher J., Rafflenbeul E., Roeher K., von Sandersleben A., Diedrichs S., Reichenspurner H., Goetz A.E. (2013). Individually optimized hemodynamic therapy reduces complications and length of stay in the intensive care unit: A prospective, randomized controlled trial. Anesthesiology.

[32] Cannesson M., Ramsingh D., Rinehart J., Demirjian A., Vu T., Vakharia S., Imagawa D., Yu Z., Greenfield S., Kain Z. (2015). Perioperative goal-directed therapy and postoperative outcomes in patients undergoing high-risk abdominal surgery: A historical-prospective, comparative effectiveness study. Crit. Care.

[33] Vallet B., Blanloeil Y., Cholley B., Orliaguet G., Pierre S., Tavernier B. (2013). Guidelines for perioperative haemodynamic optimization. Ann. Fr. Anesth. Reanim..

[34] Cannesson M., Pestel G., Ricks C., Hoeft A., Perel A. (2011). Hemodynamic monitoring and management in patients undergoing high risk surgery: A survey among North American and European anesthesiologists. Crit. Care.

